# Advances in biotechnology and breeding innovations in China’s marine aquaculture

**DOI:** 10.1007/s44307-024-00043-7

**Published:** 2024-10-18

**Authors:** Wenteng Xu, Yang Liu, Ming Li, Sheng Lu, Songlin Chen

**Affiliations:** 1 State Key Laboratory of Mariculture Biobreeding and Sustainable Goods, Yellow Sea Fisheries Research Institute, Chinese Academy of Fishery Sciences, Qingdao, 266071 Shandong China; 2 Laboratory for Marine Fisheries Science and Food Production Processes, Qingdao Marine Science and Technology Center, Qingdao, 266237 Shandong China

**Keywords:** Biotechnology, Marine aquaculture, Genome sequencing, Molecular marker, Genomic selection, Genome editing

## Abstract

Biotechnology is the key driving force behind the sustainable development of aquaculture, as biological innovation would significantly improve the capabilities of aquatic breeding and achieve independent and controllable seeding sources to ensure food safety. In this article, we have analyzed the current status and existing problems of marine aquaculture in China. Based on these data, we have summarized the recent (especially the last 10 years) biotechnological innovation and breeding progress of marine aquaculture in China, including whole genome sequencing, sex-related marker screening, genomic selection, and genome editing, as well as progress of improved marine fish varieties in China. Finally, the perspectives in this field have been discussed, and three future countermeasures have been proposed.

## Introduction

### The current situation

Marine aquaculture is an important industry for ensuring food safety in China. Over the past 40 years, China's marine aquaculture has experienced significant development, transitioning from land-based ponds, mudflats, and shallow seas to land-based factories, offshore cages, deep sea aquaculture ship. This development has resulted in the formation of “multi-varieties and multi-models” patterns. According to China Fisheries Statistical Yearbook (Bureau of Fisheries 2024), the per capita share of aquatic products in China has increased from 1.65 kg in 1950 to 50.48 kg in 2023, with marine aquaculture playing an essential role in the national food supply by providing abundant aquatic products. In 2023, China's total marine aquaculture production reached 23.95 million tons (including fish, crustacea, mollusc, algae etc.), accounting for about 41% of the country's total aquaculture production. As the world's largest marine aquaculture nation, China's marine aquaculture production has ranked first globally for 33 consecutive years (FAO reports).

### The existing problems

Despite the rapid development of marine aquaculture in China, several issues have persisted, such as slow growth, long breeding cycles, and poor disease resistance. To date, 283 new aquatic varieties have been approved in China, but most of them are freshwater species primarily focused on rapid growth, with less emphasis on disease resistance, high quality, or high feed conversion rate. Additionally, most new varieties are generated by traditional breeding techniques such as hybridization, population and family breeding. However, traditional breeding techniques are limited in the practice. For example, polygenic traits (disease-resistance) could not be effectively improved by traditional breeding technique but are suitable for whole genome selection. Therefore, the application of modern breeding biotechnology, such as whole genome selection, genome editing, and molecular design breeding techniques, holds significant potential in aquaculture (Zhou et al. 2024a, b).

This article analyzes the advancements in biotechnology and breeding innovations of marine aquaculture in China, summarizing the experience of rapid development, and more importantly, discovering the problems and challenges in the field. Based on these insights, the future countermeasures have been proposed to support construction of China into a maritime power.

## Progress in basic research of marine aquaculture in China

### Genome sequencing of marine aquatic animals

In the past decade China has achieved a number of significant milestones in the genome sequencing of marine aquatic animals, positioning itself at the forefront of marine genomic research. Since the deciphering of the Pacific oyster (*Crassostrea gigas*) genome in 2012 (Zhang et al. 2012), China has successfully sequenced the genomes of over 60 marine aquatic species. Some of these studies, combining genomic sequencing with the analysis of the formation mechanism of economic trait, have been published in top journals, showcasing China's advanced capabilities in this field. In 2014, the first fish genome, Chinese tongue sole (*Cynoglossus semilaevis*), was sequenced in China (Chen et al. 2014). Following this, scientists from the Chinese Academy of Sciences sequenced the tiger tail seahorse (*Hippocampus comes*) genome in 2016 (Lin et al. 2016). In 2017, our team directed the sequencing of the Japanese flounder (*Paralichthys olivaceus*) genome (Shao et al. 2017).

To date, over 30 marine fish genomes have been sequenced in China, including economically important species such as the large yellow croaker (*Larimichthys crocea*) (Ao et al. 2015), sea bass (*Lateolabrax maculatus*) (Shao et al. 2018), and various groupers (Table 1). Beyond fish, significant progress has been made in shellfish and crustaceans, furthering our understanding of marine biodiversity and the genetic basis of adaptability and resilience in marine species. These genomic resources are essential for analyzing key traits and advancing molecular breeding techniques, placing China at the forefront of fish genomic research.

**Table 1:** The reported genome of marine animals by Chinese scientists

	**Latin name**	**Common name**	**Order**	**Family**	**Genome size (bp)**	**Contig N50 (bp)**	**Scaffold N50 (bp)**	**Authors**
**FISH**	*Cynoglossus semilaevis*	Chinese tongue sole	Pleuronectiformes	Cynoglossidae	477.00 M	26.50 K	867.00 K	Chen et al. 2014
	*Larimichthys crocea*	large yellow croaker	Perciformes	Sciaenidae	679.00 M	63.10 K	1.03 M	Wu et al. 2014
					728.00 M	25.71 K	498.70 K	Ao et al. 2015
					723.86 M	2.83 M	/	Chen et al. 2019
	*Takifugu flavidus*	tawny puffer	Tetraodontiformes	Tetraodontidae	390.00 M	2.80 K	305.70 K	Gao et al. 2014
	*Scartelaos histophorus*	blue mudskipper	Perciformes	Gobiidae	806.00 M	8.40 K	14.30 K	You et al. 2014
	*Boleophthalmus pectinirostris*	blue-spotted mudskipper			983.00 M	20.20 K	2.31 M	
	*Periophthalmodon schlosseri*	giant mudskipper			780.00 M	16.80 K	39.10 K	
	*Periophthalmus magnuspinnatus*	giant-fin mudskipper			739.00 M	27.60 K	288.50 K	
	*Miichthys miiuy*	brown croaker	Perciformes	Sciaenidae	636.22 M	73.32 K	1.15 M	Xu et al. 2016
	*Mola mola*	sunfish	Tetraodontiformes	Molidae	730.00 M	20.00 K	9.00 M	Pan et al. 2016
	*Hippocampus japonicus*	seahorse	Syngnathiformes	Syngnathidae	501.60 M	34.70 K	1.80 M	Lin et al. 2016
	*Hippocampus erectus*	lined seahorse	Syngnathiformes	Syngnathidae	489.00 M	14.57 K	1.97 M	Lin et al. 2017a, b
	*Paralichthys olivaceus*	bastard halibut	Pleuronectiformes	Paralichthyidae	546.00 M	30.50 K	3.90 M	Shao et al. 2017
	*Lateolabrax maculatus*	spotted seabass	Perciformes	Lateolabracidae	0.62 G	31.00 K	1040.00 K	Shao et al. 2018
	*Sillago sinica*	/	Perciformes	Sillaginidae	534.00 M	/	2.60 M	Xu et al. 2018
	*Acanthopagrus schlegelii*	black porgy	Perciformes	Sparidae	688.00 M	17.20 K	7.60 M	Zhang et al. 2018
	*Dissostichus mawsoni*	Antarctic toothfish	Perciformes	Notothenioide	757.00 M	23.10 K	2.20 M	Chen et al. 2019
	*Sebastes schlegelii*	jacopever	Scorpaeniformes	Scorpaenidae	811.00 M	3.85 M	/	He et al. 2019
	*Collichthys lucidus*	light maigre	Perciformes	Sciaenidae	877.00 M	1.10 M	35.90 M	Cai et al. 2019
	*Epinephelus akaara*	Hongkong grouper	Perciformes	Epinephelidae	1.13 G	5.25 M	46.03 M	Ge et al. 2019
	*Oplegnathus fasciatus*	striped knifejaw	Perciformes	Oplegnathidae	778.70 M	2.10 M	33.50 M	Xiao et al. 2019
	*Epinephelus lanceolatus*	giant grouper	Perciformes	Epinephelidae	1.09 G	119.90 K	46.20 M	Zhou et al. 2019a, b
					1.13 G	1.46 M	1.51 M	Wang et al. 2019a, b
					1.06 G	24.33 M	45.21 M	Yang et al. 2023
	*Takifugu bimaculatus*	fugu bimaculatus	Tetraodontiformes	Tetraodontidae	404.21 M	1.31 M	16.78 M	Zhou et al. 2019a, b
	*Trachinotus ovatus*	golden pompano	Perciformes	Carangidae	647.5 M	1.80 M	5.05 M	Zhang et al. 2019
		pompano	Perciformes	Carangidae	644.45 Mb (female)	2.98 M	13.30 M	Luo et al. 2023
					652.12 Mb (male)	666.41 K	9.46 M	
	*Thamnaconus modestus*	black scraper	Tetraodontiformes	Monacanthidae	474.31 M	22.46 M	23.05 M	Bian et al. 2020
	*Scophthalmus maximus*	turbot	Pleuronectiformes	Scophthalmidae	584.00 M	16.50 K	5.90 M	Xu et al. 2020
	*Plectropomus leopardus*	leopard coral grouper	Perciformes	Serranidae	881.55 M	856.00 K	34.15 M	Zhou et al. 2020a, b
					787.06 M	1.14 M	33.85 M	Yang et al. 2020
					945.00 M	1.42 M	40.04 M	Wang et al. 2020
					849.74 M	35.59 M	38.02 M	Han et al. 2023
	*Trinectes maculatus*	triple fin spotted sole	Pleuronectiformes	Soleidae	455.73 M	99.00 K	364.00 K	Lü et al. 2021
	*Brachirus orientalis*	oriental reed sole	Pleuronectiformes	Soleidae	634.00 M	294.00 K	5.48 M	
	*Paraplagusia blochi*	short hooked whiskers	Pleuronectiformes	Soleidae	562.00 M	36.00 K	175.00 K	
	*Chascanopsetta lugubris*	/	Pleuronectiformes	Soleidae	590.00 M	14.00 K	64.00 K	
	*Oplegnathus punctatus*	spotted knifejaw	Perciformes	Oplegnathidae	831.00 M	/	29.92 M	Li et al. 2021a, b, c
	*Epinehelus moara*	kelp grouper	Perciformes	Epinephelidae	1.08 G	2.22 M	44.93 M	Zhou et al. 2021
	*Scartelaos histophorus*	blue mudskipper	Perciformes	Gobiidae	869.5 M	9.02 M	35.28 M	Li et al. 2022
	*Verasper variegatus*	spotted halibut	Pleuronectiformes	Pleuronectidae	545.34 M	14.45 M	24.76 M	Xu et al. 2022a, b
	*Epinephelus cyanopodus*	purple rockcod	Perciformes	Epinephelidae	998.82 M	5.85 M	41.98 M	Cao et al. 2022
	*Epinephelus tukula*	potato grouper	Perciformes	Epinephelidae	1.13 G	5.37 M	44.73 M	Wang et al. 2022a, b, c
	*Seriola aureovittata*	yellowtail kingfish	Perciformes	Carangidae	649.86 M	22.21 M	28.35 M	Li et al. 2022
	*Epinephelus coioides*	orange-spotted grouper	Perciformes	Epinephelidae	1.02 G	2.49 M	2.47 M	Li et al. 2022
	*Larimichthys polyactis*	little yellow croaker	Perciformes	Sciaenidae	706.15 M	1.20 M	4.52 M	Xie et al. 2023a, b
	*Decapterus maruadsi*	Japanese scad	Perciformes	Carangidae	723.69 M	24.67 M	24.67 M	Chen et al. 2024
**SHRIMP and CRAB**	*Litopenaeus vannamei*	Pacific white shrimp	Decapoda	Penaeidae	1.66 G	57.65 K	605.56 K	Zhang et al. 2019
	*Portunus trituberculatus*	swimming crab	Decapoda	Portunidae	1.00 G	4.12 M	21.80 M	Tang et al. 2020
	*Scylla paramamosain estampador*	mud crab	Decapoda	Portunidae	1.55 G	191 K	/	Zhao et al. 2021a, b
	*Portunus trituberculatus*	swimming crab	Decapoda	Portunidae	1.2 G	108.7 K	15.6 M	Lv et al. 2022
	*Fenneropenaeus chinensis*	Chinese shrimp	Decapoda	Penaeidae	1.47 G	472.84 K	36.87 M	Wang et al. 2022a, b, c
	*Marsupenaeus japonicus*	kuruma shrimp	Decapoda	Penaeidae	1.54 G	229.97 K	38.27 M	Ren et al. 2022
**MOLLUSC**	*Crassostrea gigas*	Pacific oyster	Ostreida	Ostreidae	559 M	19.4 K	401 K	Zhang et al. 2012
	*Chlamys farreri*	Zhikong scallop	Pectinida	Pectinidae	779.9 M	21.5 K	602 K	Li et al. 2017
	*Patinopecten yessoensis*	Yesso scallop	Pectinida	Pectinidae	988 M	38 K	804 K	Wang et al. 2017
	*Argopecten purpuratus*	Peruvian scallop	Pectinida	Pectinidae	724.78 M	80.11 K	1.02 M	Li et al. 2018a, b
	*Sinonovacula constricta*	razor clam	Cardiida	Solecurtidae	1.22 G	976.94 K	65.93 M	Ran et al. 2019
	*Scapharca broughtonii*	blood clam	Arcoida	Arcidae	885 M	1.8 M	45 M	Bai et al. 2019
	*Ruditapes philippinarum*	Manila clam	Venerida	Veneridae	1.12 G	28.11 K	345.K	Yan et al. 2019
	*Haliotis rubra*	Southern Hemisphere blacklip abalone	Lepetellida	Haliotidae	/	/	/	Gan et al. 2019
	*Crassostrea hongkongensis*	Hongkong oyster	Ostreida	Ostreidae	610 M	2.57 M	4.99 M	Peng et al. 2020
	*Argopecten irradians*	Atlantic bay scallop	Pectinida	Pectinidae	835.7 M	78.54 K	1.53 M	Liu et al. 2020
	*Sinonovacula constricta*	razor clam	Cardiida	Solecurtidae	1.33 G	678.86 K	57.99 M	Dong et al. 2020
	*Octopus sinensis*	East Asian common octopus	Octopoda	Octopodidae	2.72 G	490.0 K	105.9 M	Li et al. 2020a, b
	*Mytilus coruscus*	hard-shelled mussel	Mytilida	Mytilidae	1.57 G	/	/	Yang et al. 2021
	*Mercenaria mercenaria*	hard clam	Venerida	Veneridae	1.79 G	1.77 M	91.38 M	Song et al. 2021
	*Crassostrea ariakensis*	estuarine oyster	Ostreida	Ostreidae	613.89 M	6.97 M	62.26 M	Li et al. 2021a, b, c
	*Crassostrea ariakensis*	estuarine oyster	Ostreida	Ostreidae	662.9 M	5.9 M	66.34 M	Wu et al. 2021
	*Tegillarca granosa*	blood clam	Arcoida	Arcidae	812.61 M	599.92 K	/	Bao et al. 2021
	*Ruditapes philippinarum*	Manila clam	Venerida	Veneridae	1.41 G	1.83 K	/	Xu et al. 2022a, b
	*Placopecten magellanicus*	Atlantic deep-sea scallop	Pectinida	Pectinidae	1458 M	/	66.33 M	Han et al. 2022
	*Placopecten magellanicus*	Atlantic deep-sea scallop	Pectinida	Pectinidae	1.78 G	6.53 M	86.71 M	Xie et al. 2023a, b
	*Crassostrea angulata*	Portuguese oyster	Ostreida	Ostreidae	624 M	/	60.48 M	Teng et al. 2023
**SEACUCUMBER**	*Apostichopus japonicus*	sea cucumber	Aspidochirotida	Stichopodidae	804 M	190.27 K	486.65 K	Zhang et al. 2017
					952 M	45 K	196 K	Li et al. 2018a, b
					671.60 M	17.20 M	29.65 M	Sun et al. 2023
	*Holothuria leucospilota*	tropical sea cucumber	Holothuroidea	Holothuriidae	1.39 G	55.5 M	56.1 M	Chen et al. 2023
	*Holothuria scabra*	tropical sea cucumber	Holothuroidea	Holothuriidae	1.19 G	3.15 M	53.52 M	Zhong et al. 2024

### Oyster genome (the first mollusc genome in China)

In 2012, scientists from Chinese Academy of Sciences made breakthrough for uncovering the adaptation mechanism of oyster to intertidal environments. Utilizing next-generation sequencing technologies, the team constructed a comprehensive oyster genome map. Their findings revealed the high polymorphism of the oyster genome, a significant proportion of repetitive sequences, and active transposons. Through integrated transcriptomic and proteomic analyses, the study identified a series of genes that have undergone notable expansions, associated with the oyster's resilience to environmental stressors, highlighting the molecular basis of their adaptability.

Globally distributed, oysters are emblematic of the extreme intertidal habitat and represent the highest yielding marine aquaculture species, providing an essential source of marine protein. China's oyster production accounts for three-quarters of the global output. Due to their ecological significance, economic value, and unique biological traits, oysters have become a focal point in marine biological research and a model species for studying marine organism adaptation to environmental stress. Genomic and transcriptomic analyses revealed an expansion of genes related to stress resistance. For instance, the number of heat shock protein 70 (HSP70) genes in oysters reaches 88, nearly five times the average than sea anemones, potentially enabling oysters to maintain cellular homeostasis at temperatures as high as 49°C. Additionally, the number of inhibitor of apoptosis protein (IAP) genes is 48, approximately nine times than that in other species, suggesting a complex anti-apoptotic system to allow oysters to survival at prolonged exposure to harsh conditions. For shell biomineralization mechanism, researchers discovered the involvement of various proteins in the intricate assembly and modification of shells, including the participation of hemocytes and exosomes. Notably, most shell proteins are non-secretory, indicating that the biomineralization process is far more complex than previously understood. These findings provide valuable insights into the evolutionary processes of intertidal marine organisms and marine genetic adaption in the context of increasing ocean acidification (Zhang et al. 2012). Notably, the high polymorphism and abundant repetitive sequences lead to considerable challenges for de novo genome assembly. Over 62% of the detected repeats in the oyster genome are still unclassified, underscoring the lack of genomic data from molluscan species. To address these chanllenges and achieve a more comprehensive assembly, it is advisable to use sequencing technologies capable of generating longer reads, such as PacBio's High-Fidelity (HIFI) reads or Oxford Nanopore's ultra-long reads. These advanced technologies can more effectively resolve repetitive regions and allelic variations more effectively, thereby improving the quality and completeness of the oyster genome assembly.

### Chinese tongue sole genome (the first fish genome in China)

The Chinese tongue sole is a flatfish of significant economic importance in China, with females typically being 2-4 times larger than males. Despite the species’ economic value, the males population ratio is higher, approximately 70-90%. Understanding the underlying genetic mechanisms responsible for these sex differences is crucial for both scientific and commercial purposes.

In 2014, whole genome sequencing annotated 21,516 genes, including a comprehensive assembly of the Z and W chromosomes (ZW sex determination). Among the identified genes, *dmrt1* has been suggested as the male-determining gene. This gene is exclusively expressed in the testis and plays a critical role during the sex differentiation period. When genetic females sex-reverse to phenotypic males, *dmrt1* expression is significant upregulated. The expression of *dmrt1* is directly correlated with the slow growth and smaller size observed in males. These findings have elucidated the sex determination mechanism in the Chinese tongue sole and underscored the significant impact of *dmrt1* on growth regulation, paving the way for manipulating *dmrt1* expression to improve yields (Chen et al. 2014). While many differentially expressed genes and candidate genes have been identified, their functional roles and regulatory mechanisms in adaptation and sex determination have not been fully validated. It is necessary to conduct functional studies, including gene knockout/knockdown experiments and overexpression studies, to validate the roles of candidate genes in adaptation to the benthic lifestyle and sex determination.

### Japanese flounder genome (Elucidating the metamorphosis mechanism)

The Japanese flounder is a commercially valuable flatfish, notable for its complex metamorphosis process. This process is critical for proper development, as abnormalities can result in fry malformation and atypical body coloration, impacting the viability and market value of the fish.

The whole genome sequencing of the Japanese flounder has provided significant insights into its metamorphosis mechanism. Traditionally, it was believed that metamorphosis was primarily controlled by thyroid hormone. However, our study revealed a more complex regulatory mechanism involving retinoic acid, which can inhibit eye migration and metamorphosis in flounder. This discovery demonstrated that metamorphosis is regulated by the dual antagonistic actions of thyroid hormone and retinoic acid.

These findings offer a more comprehensive understanding that challenges traditional metamorphosis mechanism and provides new perspective for the strategies to prevent malformations and improve the fry quality (Shao et al. 2017). However, the majority of the findings are based on genomic and transcriptomic analyses. Additional functional validation, especially using in vivo experiments, is needed to confirm the roles of identified genes and pathways.

### Seahorse genome (Body Type Adaptation and Reproductive Evolution)

Seahorses are a unique type of fish with a distinctive body type, which are worldwide distributed in the oceans. They serve as crucial environmental indicators in marine ecosystems and are often referred to as "marine ginseng" due to their exceptional medicinal properties. The unique male parenting and mating behaviors of seahorses have long intrigues scientists.

Research based on the seahorse genome has explored the formation of the brood pouch and the male pregnancy process have been investigated, providing insights into male seahorse parenting. For the first time, this research has elucidated he unique evolutionary mechanisms behind the seahorse's body type, enhancing our understanding of the evolutionary status and environmental adaptability of marine fish. In this study, seahorses are found to be the fastest-evolving species among sequenced fish genomes. Genes associated with environmental adaptation display significant shrinkage. For example, seahorses have only 26 olfactory receptor genes (ORs), compared to 60 to 169 in other fish species. The Syngnathidae family is the only known animal group exhibiting "male parenting." The researchers discovered that the novel gene *pastn*, associated with the brood pouch, has undergone amplification and exhibits high expression. While similar structures and gene combinations are present in the *c6ast* gene family of swordtails, the *pastn* gene in seahorses shows an "independent evolution" pattern. The gene duplication and selective expression mechanism of *pastn* genes have novel functional characteristics in the development of the brood pouch and the male pregnancy process, providing a foundation for understanding male seahorse parenting. Another noteworthy aspect of this study is the investigation into the mechanism of ventral fin degeneration in seahorses. The researchers found that seahorses lack the *tbx4* gene. The knockout of this gene in zebrafish led to a complete loss of the ventral fin, without affecting other body-related characteristics. This finding confirms that the loss of the *tbx4* gene is the crucial reason for the absence of ventral fins in seahorses (Lin et al. 2016). The current study provides valuable insights into the genetics underlying the specialized morphology and reproductive system of seahorses. However, further research is needed to address remaining questions, particularly regarding the mechanisms driving the higher evolutionary rates, gene losses, and detailed molecular interactions and regulatory mechanisms of candidate genes. Through a combination of experimental validation, comparative genomics, and functional annotation, future studies can deepen our understanding of the unique evolutionary adaptations of seahorses.

### Pacific white shrimp genome (the first penaeid shrimp genome in China)

The Pacific white shrimp (*Litopenaeus vannamei*), also known as the whiteleg shrimp, is a significant economic shrimp species widely distributed across various aquatic environments. It holds immense value in the aquaculture industry, particularly due to its rapid growth rate and adaptability to various farming conditions.

A research team from the Institute of Oceanology, Chinese Academy of Sciences, has successfully deciphered the complex genome of *L. vannamei*, marking a significant milestone in the field of aquaculture genomics (Zhang et al. 2019). This achievement, published in the journal Nature Communications, provides the first high-quality reference genome assembly for the species. The study not only sheds light on the shrimp's unique adaptations to its benthic habitat and its frequent molting process but also lays a solid foundation for shrimp genomic breeding and molecular improvement programs.

### Sea cucumber genome (the first sea cucumber genome)

The *Apostichopus japonicus*, commonly known as sea cucumber, is a remarkable species belonging to the Echinodermata phylum, renowned for its distinct morphology and evolutionary position bridging the gap between invertebrates and vertebrates. As a significant marine economic resource, it boasts nutritional and medicinal values globally.

In the study published in PLoS Biology, researchers from the Chinese Academy of Sciences' Oceanography Institute presents the first high-resolution reference genome of the sea cucumber, unraveling the molecular underpinnings of its unique morphological evolution and remarkable regenerative potential (Zhang et al. 2017). This seminal work not only advances our understanding of echinoderm evolution and tissue regeneration mechanisms but also lays a solid foundation for regenerative medicine applications and genetic breeding programs of sea cucumbers.

## Progress in marine animal breeding biotechnology in China

### Molecular marker-assisted sex control technology

Sex-specific markers are essential tools for studying sex determination mechanisms and controlling sex, especially given the pronounced sexual dimorphism in many marine species (Mank 2009).

Chinese tongue sole, a typical sexually dimorphic marine fish, has female that grow 2-4 times faster than males (Wang et al., 2021). In 2007, the first female-specific AFLP molecular marker (M1) was developed for Chinese tongue sole, marking the first fish sex-specific marker in China (Chen et al. 2007). This marker amplified a specific DNA band in all female individuals but not in any male individuals, demonstrating the effectiveness of the female-specific marker and PCR technology in Chinese tongue sole. However, the M1 marker is unable to screen and WW super females (produced by gynogenesis) from regular ZW females, while identifying WW super females is critical for producing all-female Chinese tongue sole offspring. In 2014, sex-linked microsatellite markers CseF-SSR1 (M2) was identified, which could distinguish ZW and WW individuals (Chen et al. 2012). These markers hold significant application value and broad prospects in identifying broodstock for breeding, gynogenetic fry identification, sex control, and the cultivation of high-female or all-female offspring.

Despite the fundamental role of sex-specific markers in fish sex determination mechanisms and sex-controlled breeding, the development and identification of molecular markers such as RFLP, RAPD, AFLP, and SSR have low throughput and are labor-intensive. With the rapid advancement in genome sequencing, sex-specific markers have been identified in over thirty marine species, including Chinese tongue sole (*C. semilaevis*) (Chen et al. 2007, Chen et al. 2012, Song et al. 2012, Liu et al. 2014, Dong et al. 2016a, b, c), rock bream (*Oplegnathus fasciatus*) (Xu et al. 2013), large yellow croaker (*Larimichthys crocea*) (Lin et al. 2018, Lin et al. 2017a, b; Yu et al. 2022), yellow drum (*Nibea albiflora*) (Sun et al. 2018)*, * spotted knifejaw (*Oplegnathus punctatus*) (Li et al. 2020a, b)*,* spotted halibut (*Verasper variegatus*) (Ma et al. 2010, Zheng et al. 2024)*,* golden pompano (*Trachinotus ovatus*) (Guo et al. 2021)*,* farmed ayu (*Plecoglossus altivelis*) (Li et al. 2021a, b, c)*,* sea cucumber (*Stichopus monotuberculatus*) (Wu et al. 2022)*,* sea urchin (*Mesocentrotus nudus*) (Cui et al. 2021)*,* and swimming crab (*Portunus trituberculatus*) (Lv et al. 2018) (Table 2).

**Table 2:** The Reported marine fish sex-specific molecular markers by Chinese scientists

Common name	Latin name	Sex determination type	Marker type	Authors
Chinese tongue sole	*Cynoglossus semilaevis*	ZW-ZZ	AFLP	Chen et al. 2007
spotted halibut	*Verasper variegatus*	ZW-ZZ	AFLP	Ma et al. 2010
Chinese tongue sole	*Cynoglossus semilaevis*	ZW-ZZ	SSR	Chen et al. 2012
Chinese tongue sole	*Cynoglossus semilaevis*	ZW-ZZ	SSR	Song et al. 2012
rock bream	*Oplegnathus fasciatus*	X1X2Y	SSR	Xu et al. 2013
Chinese tongue sole	*Cynoglossus semilaevis*	ZW-ZZ	SCAR	Liu et al. 2014
Chinese tongue sole	*Cynoglossus semilaevis*	ZW-ZZ	DNA Fragment	Dong et al. 2016a, b, c
large yellow croaker	*Larimichthys crocea*	XX-XY	SNP	Lin et al. 2017a, b
large yellow croaker	*Larimichthys crocea*	XX-XY	DNA Fragment	Lin et al. 2018
yellow drum	*Nibea albiflora*	XX-XY	DNA Fragment	Sun et al. 2018
spotted knifejaw	*Oplegnathus punctatus*	X1X2Y	DNA Fragment	Li et al. 2020a, b
golden pompano	*Trachinotus ovatus*	ZZ-ZW	SNP	Guo et al. 2021
farmed ayu	*Plecoglossus altivelis*	XX-XY	DNA Fragment	Li et al. 2021a, b, c
large yellow croaker	*Larimichthys crocea*	XX-XY	DNA Fragment	Yu et al. 2022
spotted halibut	*Verasper variegatus*	ZW-ZZ	DNA Fragment	Zheng et al. 2024
sea cucumber	*Stichopus monotuberculatus*	XX-XY	DNA Fragment	Wu et al. 2022
sea urchin	*Mesocentrotus nudus*	ZW-ZZ	SNP	Cui et al. 2021
swimming crab	*Portunus trituberculatus*	XX-XY	DNA Fragment	Lv et al. 2018

Currently, sex-specific molecular markers have been applied in the sex-controlled breeding of various well-researched marine animals, yielding significant results. For example, in the breeding process of the high-quality Chinese tongue sole, a rapid genetic sex identification technology for male candidate broodstock was established in the controlled aquaculture environment, eliminating pseudo-males and ensuring that the female proportion of the progeny exceeded 40%. Subsequently, by combining family selection and genomic selection breeding techniques, a new high-female, fast-growing, disease-resistant Chinese tongue sole strain, "Tayou No. 1", was developed. This strain exhibited a 30.9% increase in resistance to *Vibrio harveyi* infection, a 17.7% increase in average body weight at 18 months of age, and a 15.7% increase in average aquaculture survival rate (Lu et al. 2022).

As of 2023, China has approved 283 new aquaculture cultivars, including 11 all-female/male or high-female/male cultivars. Among them, sex-specific molecular markers played a crucial role in the sex control of the high-female Chinese tongue sole cultivar "Tayou No. 1" and the all-female yellow croaker variety "Quanci No. 1".

### Genomic selection

As the quality of life improves, people's expectations for aquatic foods have risen, including lower fishery medicine residues, better animal welfare, and superior meat quality. Therefore, breeding varieties with excellent traits, such as high disease resistance, rapid growth performance, abundant unsaturated fatty acid content, broad environmental tolerance, are essential to meet these requirements. Since most of these economic traits are polygenic, it is challenging to accurately select promising candidates for breeding schemes using traditional breeding methods, including mass breeding, crossbreeding, and family selection based on the best linear unbiased prediction (BLUP). Genomic selection (GS), introduced by Meuwissen et al. in 2001, is a revolutionary concept that utilizes the genetic markers across the entire genome to estimate additive genetic effects and select individuals with superior genomic estimated breeding values (GEBV). Due to its high prediction accuracy, reduction of breeding cycles, and excellent performance in predicting breeding values of complex traits, GS has been rapidly developed in the field of aquatic breeding since 2014 and has become a mainstream breeding method for improving economic important traits in aquatic species in China (Liu et al. 2018).

The reliability of GS was first assessed in Atlantic salmon (*Salmo salar*) in 2014 abroad (Ødegård et al. 2014). Subsequently, GS was employed for genetic improvements in other economically important species, such as rainbow trout (*Oncorhynchus mykiss*) (Vallejo et al. 2016), Pacific oyster (*Crassostrea gigas*) (Gutierrez et al. 2018), and Pacific white shrimp (*Litopenaeus vannamei*) (Lillehammer et al. 2020). With the flourishing of genome analysis and molecular biotechnology, the research and applications of GS have flourished in mollusc, fish, and shrimp since 2016 in China, promoting the development of molecular breeding technology and selection new varieties. At present, around 34 GS-related studies have been published in China (Table 3), and eight new varieties have been developed by comprehensively using family selection and GS, including Japanese flouner (*Paralichthys olivacaus*) “Pingyou No.2”, Chinese tongue sole (*Cynoglossus semilaevis*) “Tayou No.1”, Zhikong scallop (*Chlamys farreri*) “Penglaihong No.2”, “Penglaihong No.3”, and “Penglaihong No.4”, and Yesso scallop (*Patinopecten yessoensis*) “Haiyifeng 11”, “Haiyifeng 12”. The successful application of GS in developing these new varieties not only demonstates the broad prospects of GS in genetic improvements but also sets the trends for molecular breeding technology economically in Chinese aquatic breeding (Zhou et al. 2024a, b).

**Table 3:** The genomic selection research in marine aquaculture in China

Category	Species	Trait	Authors
FISH	*Acipenser gueldenstaedtii*	Body weight	Song et al. 2022
	*Cynoglossus semilaevis*	*Vibrio harveyi* resistance	Lu et al. 2021
	*Epinephelus coioides*	Ammonia tolerance	Shan et al. 2021
		Body weight and ammonia tolerance	Shan et al. 2023
	*Larimichthys crocea*	Eviscerated weight, eviscerated weight/whole body weight ratio	Dong et al. 2016a, b, c
		Body weight, body length, and n-3 highly unsaturated fatty acids in muscle	Dong et al. 2016a, b, c
		*Cryptocaryon irritans* resistance	Zhao et al. 2021a, b
		*Pseudomonas plecoglossicida* resistance	Bai et al. 2022
		Adapting to high plant protein diet	Ke et al. 2022
		*C. irritans* resistance	Wang et al. 2022a, b, c
		Swimming performance and disease resistance (*C. irritans*, *V. alginolyticus*, and *P. plecoglossicida*)	Zeng et al. 2023
		White gill disease (WGD) resistance	Zhou et al. 2023
		Hypoxia tolerance	Ding et al. 2024
	*Lateolabrax maculatus*	Body weight, body height, total length and body length	Zhang et al. 2023
	*Nibea albiflora*	Body length, swimming bladder index, swimming bladder weight, body thickness, body height, body length/body height ratio, and gonad weight index	Liu et al. 2019
	*Oncorhynchus mykiss*	Carcass fat content	Hu et al. 2019
	*Oplegnathus fasciatus*	Body weight, total length, and body depth	Gong et al. 2021
	*Paralichthys olivaceus*	*Edwardsiella tarda* resistance	Liu et al. 2018
		*Edwardsiella tarda* resistance	Lu et al. 2020
	*Plectropomus leopardus*	*V. harveyi* resistance	Lu et al. 2023
MOLLUSC	*Argopecten irradians irradians*	Shell length, shell height, shell width, and whole weight	Zhu et al. 2021a, b
	*Chlamys farreri*	Shell length	Su et al. 2017
		Shell length, shell height, shell width, and whole weight	Wang et al. 2018
	*Crassostrea gigas*	*Vibrio alginolyticus* resistance	Yang et al. 2024
	*Haliotis discus hannai*	Heat tolerance	Liu et al. 2022a, b
		Shell length, shell width, wet weight, collagen content, taurine content, glycogen content, and total protein content	Liu et al. 2024
	*Patinopecten yessoensis*	Shell height, shell length, and shell width	Dou et al. 2016
SHRIMP	*Fenneropenaeus chinensis*	Harvest body weight	Liu et al. 2023
	*Litopenaeus vannamei*	Body length and body weight	Wang et al. 2016
		*V. parahaemolyticus* resistance	Wang et al. 2019a, b
		Feed efficiency ratio, residual feed intake, and average daily weight gain	Dai et al. 2020
		High salinity tolerance	Luo et al. 2022
		Harvest body weight	Kang et al. 2024
		Body length and abdomen length/cephalothorax length ratio	Luo et al. 2024

Genotyping is essential for both technology research and candidate selection within the GS field. Beyond genome sequencing technology, SNP array serves as a high-throughput genotyping technology capable of detecting tens of thousands of variants simultaneously. Currently, the most widely used SNP array technologies are established by Affymetrix GeneChip (Irizarry 2003) and Illumina BeadChip (Shen et al. 2005). Due to the detection being conducted on the silicon-based material, the SNP arrays based on these two technologies are referred to as solid arrays. Now, solid arrays are extensively utilized in GS research, including the Atlantic salmon 130 K arrays (Correa et al. 2015). In China, several arrays have been developed, such as the 50K array for Japanese flounder “Yuxin No.1” (Zhou et al. 2021), the 38K array for Chinese tongue sole “Solechip No.1” (Lu et al. 2021), the 600K array Ningxin-I for large yellow croaker (Zhou et al. 2020a, b) and the 55K array Ningxin-II (Zhou et al. 2022). These arrays provide efficient and convenient genotyping tools to promote the application of GS in aquatic breeding practices. However, the costs of these SNP array remain prohibitive for both GS research and breeding practices in China. In recent years, Chinese researchers have developed various genotyping technologies with independent intellectual property rights, such as HD-Marker (Lv et al. 2018), mSNP (Guo et al. 2021) and Hyper-seq (Zou and Xia 2022). These technologies leverage target-enrichment or multiplex amplification and sequencing techniques to significantly reduce genotyping costs while ensuring high accuracy and flexible design schemes. As the detection processes occur in a liquid environment, these technologies are also referred to as liquid SNP array. Currently, liquid arrays for aquatic animals, such as scallop (Cui et al. 2017, Liu et al. 2022a, b), large yellow croaker (Wang et al. 2023), abalone (Lin et al. 2023), shrimp (Liu et al., 2023) and leopard coral grouper (Zhou et al. 2024a, b), have been developed and applied (Table 4). These domestically developed liquid SNP arrays will accelerate the large-scale application of GS and advance aquatic breeding technologies into the molecular era.

**Table 4:** The SNP arrays in marine aquaculture in China

Category	Species	Density	Type	Genotyping technology	Authors
FISH	*L. crocea*	600 K	Solid	Affymetrix GeneChip (Thermo Fisher Scientific, USA)	Zhou et al. 2020a, b
		55 K	Solid	Affymetrix GeneChip (Thermo Fisher Scientific, USA)	Zhou et al. 2022
		55 K	Liquid	Targeted sequencing (MolBreeding, China)	Wang et al. 2023
	*P. olivaceus*	50 K	Solid	Affymetrix GeneChip (Thermo Fisher Scientific, USA)	Zhou et al. 2021a, b
	*C. semilaevis*	38 K	Solid	Affymetrix GeneChip (Thermo Fisher Scientific, USA)	Lu et al. 2021
	*P. leopardus*	20 K	Liquid	Captured targets sequencing (cGPS) (Higentec, China)	Zhou et al. 2024a, b
MOLLUSC	*P. yessoensis*	86 K	Liquid	High-throughput diverse marker genotyping (HD-marker)	Liu et al. 2022a, b
	*H. discushannai*	97 K	Liquid	Multiple SNP captured sequencing (GenoBaits) (MolBreeding, China)	Liu et al. 2022a, b
	*H.* spp.	427	Liquid	Multiplex PCR products sequencing (mGPS) (Higentec, China)	Lin et al. 2023
SHRIMP	*L. vannamei*	2 K	Liquid	Targeted sequencing (AmpliSeq) (Thermo Fisher Scientific, USA)	Yu et al. 2020
		40 K	Liquid	Multiple SNP captured sequencing (GenoBaits) (MolBreeding, China)	Liu et al. 2023

### Genome editing

Genome editing allows precise modification of specific target genes within the genome and has evolved through three generations (ZFN, TALEN, and CRISPR). Although genome editing has been widely reported in aquatic animals, its application in marine aquatic animals remains limited (Yang et al., 2024).

So far, marine fish that have achieved genome editing in China include Chinese tongue sole, Japanese flounder, large yellow croaker, and so on. As the first marine fish accomplished with genome editing in the world, research group in Yellow Sea Fisheries Research Institute has knocked out *dmrt1* using TALEN technology and established the homozygous mutant. The homozygous mutant male fish grew twice as fast as the normal male (Cui et al. 2017). The scientists from the Institute of Oceanography have employed electroporation technology to introduce CRISPR/Cas9 into the fertilized eggs in Japanese flounder and successfully editing *gsdf* gene (Wang et al. 2021). The research team from Xiamen University utilized CRISPR/Cas9 technology to knockout myostatin gene in large yellow croaker (Yan et al. 2022). Genome editing research abroad have been conducted in Pacific bluefin tuna, pufferfish, red sea bream, and mackerel (Higuchi et al. 2019, Kim et al. 2019, Ohama et al. 2020, Kim et al. 2021, Kawamura et al. 2022). In Pacific bluefin tuna, *ryr1b* gene mutation enables young fish to respond to collisions. The myostatin gene knockout of red sea bream and Japanese flounder has led to the increase of skeletal muscle. Genome editing of *slc45a2* gene in mackerel has produced albino mutant.

Regarding marine invertebrates, a domestic research team has made progress in microinjection of white shrimp eggs and successfully built the CRISPR/Cas9 platform, which was the first time to perform genome editing in decapods (Gui et al. 2016). The research group from Ocean University of China used CRISPR/Cas9 to mutate the *mstn* gene in Pacific oysters (Yu et al. 2019). The institute of Oceanography has established genome editing technology in sea mollusc (Huan et al. 2021). A research group in the United States achieved gene editing in Hawaiian shrimp by mutating the segment development gene hox (Serano et al. 2016). In addition, genome editing studies of Atlantic snails, sea urchins, and sea squirts have also been initiated.

### Progress in improved marine fish varieties in China

There are about 60 species of mariculture fish in China. Until now, 19 new artificially selected marine fish varieties in China have been developed, including large yellow croaker (4), Japanese flounder (4), turbot (3), Grouper (3), yellow drum (1), Chinese tongue sole (1), fugu (2), golden pompano (1). These new varieties have shown great breeding potential through genomic selection techniques. For example, Japanese flounder “Pingyou No.2”, the first fish new variety generated by genomic selection in China, has shown increased survival rate of 30% by bacterial challenging and 25% increased rearing survival rate. Another example is the first Chinese tongue sole new variety “Tayou No.1”, which has shown improvements in the challenging survival rate, rearing survival rate, and growth performance increased 31%, 16%, and 18%, respectively. Besides genomic selection, genome editing and other modern biotechnologies have been widely used to generate superior germplasm. It is expected the modern biotechnologies will play more important role in breeding practice in future.

## Challenges and perspectives

Despite the significant advancements in China over the past decade, there are still several challenges as follows:

1. Basic research requires to be strengthened, especially the lack of original breeding theories, which limits the technological development.
2. The promotion of new technologies is urgently required, as the cultivation of new varieties mainly adopts traditional breeding techniques, which is difficult to improve complex economic traits (e.g. disease resistance).
3. The management policies are inadequate. Taking the genome editing as example the United States, Japan, Argentina, Australia, and the United Kingdom have already approved the commercialization of genome edited product, China needs to issue the relevant policy to promote applications of genome editing.

It is widely recognized that the ocean has great potential for the world food supply. To fully address the challenge and release the potential, we have the following suggestions.

1. Dissection of the genetic basis and regulatory mechanism of important traits.

Drawing gnome and pan-genome map of the marine aquatic animals, analyzed the formation and evolution mechanisms of critical traits such as growth, gender, disease resistance, stress resistance, and quality, elucidation of the mechanisms of phenotype-genotype-environment interaction.

(2) Establishment of efficient and precise modern breeding technologies.

Expanding the application scale of sex-specific markers and sex control techniques; establishment of genome selection breeding technology for multiple traits; development of efficient genome editing breeding technology in marine aquatic animals, and exploration of molecular design and intelligent breeding system.

(3) Creation of breakthrough new marine varieties.

The future trend is to cultivate new aquatic varieties simultaneously with strong disease resistance, high yield, and excellent quality through genome selection. Genome editing will be employed to generate fast growing and high-quality varieties; By combine traditional technologies such as hybridization and family selection with modern technologies genome selection and gene editing, we aim to cultivate breakthrough new varieties with multiple desired traits, which provide the independent and controllable core aquatic seeding sources and in turn ensure seed industry and food security in China.

## Data Availability

All data generated or analysed during this study are included in this published article.
